# Laparoscopic spleen-preserving hilar lymph node dissection through pre-pancreatic and retro-pancreatic approach in patients with gastric cancer

**DOI:** 10.1186/s12935-016-0312-7

**Published:** 2016-06-29

**Authors:** Liansheng Zheng, Ce Zhang, Da Wang, Qi Xue, Xiaoping Liu, Ke-Jian Zhou, Hao Liu, Guoxin Li

**Affiliations:** Department of General Surgery, Nanfang Hospital, Southern Medical University, No.1838, North Guangzhou Avenue, Guangzhou, 510515 Guangdong Province China; Department of General Surgery, Tumor Hospital of Baotou, Baotou, 014030 Inner Mongolia Autonomous Region China; The Key Laboratory of Cancer Prevention and Intervention, Department of Surgical Oncology, China National Ministry of Education, The Second Affiliated Hospital of Zhejiang University School of Medicine, 88 Jie-Fang Rd, Hangzhou, 310009 Zhejiang Province China

**Keywords:** Gastric cancer, Laparoscopy, Splenic hilum, Lymph node dissection

## Abstract

**Background:**

The conventional radical resection of proximal gastric cancer is even more risky when performed laparoscopically, though this technique is widely used in gastrointestinal surgery and is accepted as the superior method. This paper explores the feasibility of laparoscopic spleen-preserving hilar lymph node dissection using a retro-pancreatic approach for the treatment of proximal gastric cancer.

**Methods:**

Two cadavers were dissected for examination of and the pre-pancreatic and retro-pancreatic spaces. Following the dissection of the cadavers, ten live patients with proximal gastric cancer from May 2008 to May 2013 at Nanfang Hospital, Guangzhou, China, were given total gastrectomy and adjuvant splenic hilar lymph node clearance through pre-pancreatic and retro-pancreatic approach on the precondition of preserving the pancreas and spleen. The clinicopathologic characteristics, as well as the intraoperative and postoperative variables affecting the procedure, were observed and analyzed.

**Results:**

Anatomy of the space anterior and posterior to the pancreas in the two cadavers demonstrated the feasibility of pre-pancreatic and retro-pancreatic approach. The surgeries were all successfully performed laparoscopically; conversion to laparotomy was not necessary for any of the ten patients. The overall mean operative time was 243.6 ± 45 min. The mean estimated blood loss was 232 ± 80 ml. At the time of follow-up (median 12 months post-surgery), there had been neither local recurrence nor mortality in any of the patients.

**Conclusion:**

Laparoscopic spleen- and pancreas-preserving splenic hilar lymph node dissection during total gastrectomy, using both pre-pancreatic and retro-pancreatic approaches, is indicated as a safe and feasible method for the treatment of proximal gastric cancer.

## Background

Gastric cancer is the second most common cause of all cancer-related deaths worldwide [1, 2]. Proximal gastric cancer has a higher annual incident rate than all other types of gastric carcinoma [3]. Gastric cancer is not usually detected until it has reached an advanced stage. Total gastrectomy with D2 lymph node dissection is a standard course of treatment for individuals with advanced proximal cancer. In accordance with the *Japanese Gastric Cancer Treatment Guidelines* [4], splenic hilar lymph nodes are considered part of station 2 for radical resection of advanced proximal gastric cancer, which validates the need to dissect lymph nodes at the splenic hilum [5].

The conventional radical resection of proximal gastric cancer generally includes dissection of the stomach, the pancreatic tail, and the spleen [6]. It may also consist of resection of the stomach and spleen, but the pancreas will be preserved [7, 8]. However, this method not only fails to improve the survival rate of patients, but it also increases instances of postoperative complications and mortality. For example, this conventional method results in higher incidences of pancreatic fistula, acute pancreatitis, abdominal abscess, postoperative diabetes, and postoperative hemorrhage [9–13]. The conventional radical resection of proximal gastric cancer is even more risky when performed laparoscopically, though this technique is widely used in gastrointestinal surgery and is accepted as the superior method. Based on this information, we evaluated the anatomy of the cadavers and used laparoscopic techniques on the live patients to identify an optimal surgical method for preserving the functions of the pancreas and spleen while reducing postoperative complications and mortality rates.

## Methods

### Cadaver dissection

Two cadavers, a female and a male, were provided by Southern Medical University in Guangzhou, China, for use in the study. The cadavers were preserved at −20 °C. Next, they were defrosted and dissected at 4 °C. A digital camera (EOS 20D Canon, Tokyo, Japan) was used to record the observations.

### Live observation

From May 2008 to May 2013, 256 cases of proximal gastric cancer were diagnosed by gastroscopic biopsy at Nanfang Hospital, Guangzhou, China. Ten of these cases were chosen for observation in the present study. The selection criteria were as follows: (i) the carcinoma was located in the upper or middle third of the stomach; (ii) no obvious distal metastasis was present preoperatively; (iii) the patient showed enlargement of the splenic hilar lymph nodes or carcinoma invasion to the pancreas or spleen; and (iv) the patient had no severe comorbidities. Informed consent was obtained from each of the participants. According to Cancer Staging Manual (7th edition) issued by the American Joint Committee on Cancer (AJCC), TNM of these ten patients were 1 case of IB stage, 3 cases of II stage, 4 cases of IIIA stage and 2 cases of IIIB stage (Table 1).

**Table 1:** Patient characteristics and surgical outcomes

Characteristics	Data
The total number of patients	10
Age (years)	
≤50	3 (30 %)
>50	7 (70 %)
Gender	
Male	7 (70 %)
Female	3 (30 %)
The depth of tumor invasion	
T2	4 (40 %)
T3	6 (60 %)
TNM stage	
IB (T2N0M0)	1 (10 %)
II (T2N1M0)	3 (30 %)
IIIA (T3N1M0)	4 (40 %)
IIIB (T3N2M0)	2 (20 %)
The average hospitalization days	11.5 ± 3
Mean operating time	243.6 ± 45
Mean bleeding volume	232 ± 80
Lymphatic metastasis	
N0	1 (10 %)
N1	7 (70 %)
N2	2 (20 %)

A surgical group with experience in laparoscopic gastric cancer surgery performed the surgeries. After general anesthesia, the patient was placed in the reverse Trendelenburg position. The surgeon stood at the patient’s left side, with the first assistant at the patient’s right and the camera operator between the patient’s legs. Five holes were made in the abdominal wall, and CO_2_ pressure was maintained at 12–14 mmHg. Surgery proceeded using the pre-pancreatic and retro-pancreatic approach.

## Results

### The results of cadaver dissection

**Fig. 1:**
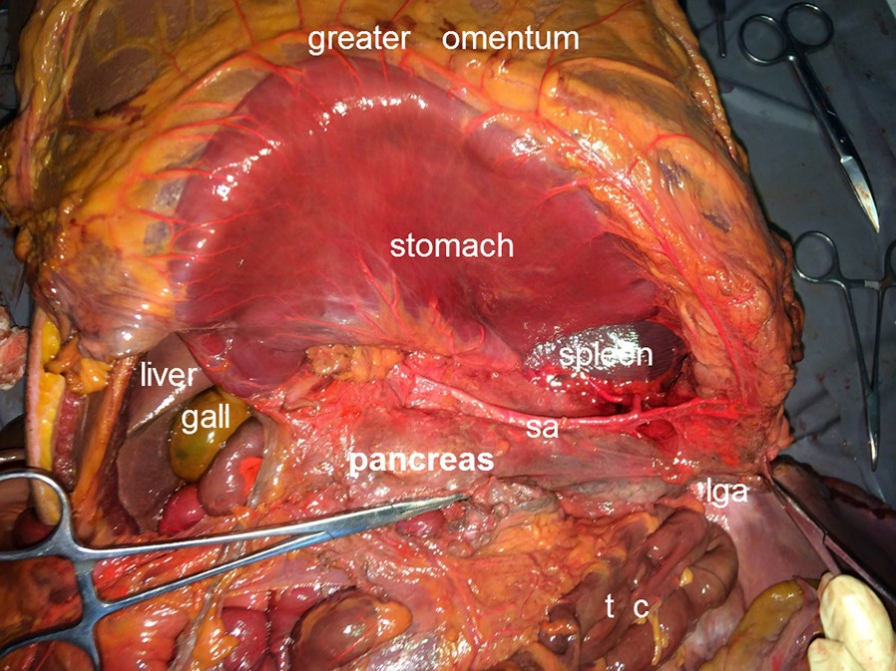
The pre-pancreatic space. *sa* splenic artery; *lga* left gastroepiploic artery; *tc* transverse colon

A midline abdominal incision was performed for complete exposure. In conformity with the laparoscopic surgical approach for gastric cancer, the anterior leaf of the transverse mesocolon was incised and the pre-pancreatic space was exposed (Fig. 1). The anterior surface of the pancreas, which is relatively large, forms the posterior wall of the lesser omental bursa and creates close contact with the posterior gastric wall and lesser curvature. The pancreatic neck is extended leftward to form the pyramid-shaped pancreatic body. The terminal of the pancreatic body tapers and runs into the two layers of the peritoneum of the splenorenal ligament, which is called the pancreatic tail and ends at the anterior splenic hilum. The anterior lobe of the transverse mesocolon was isolated up to the root of the transverse mesocolon, revealing the two layers of the peritoneum, attached along the inferior margin of the pancreas and respectively covering the anterior surface and inferior surface of the pancreatic body. The upper layer of the transverse mesocolon root ran upward to wrap around the anterior side of the pancreas and continued upward to constitute the back wall of the omental bursa, whereas the lower layer of the transverse mesocolon root stretched backward to cover the inferior side of the pancreas and flipped to the retroperitoneal wall at the top of the omental bursa.

**Fig. 2:**
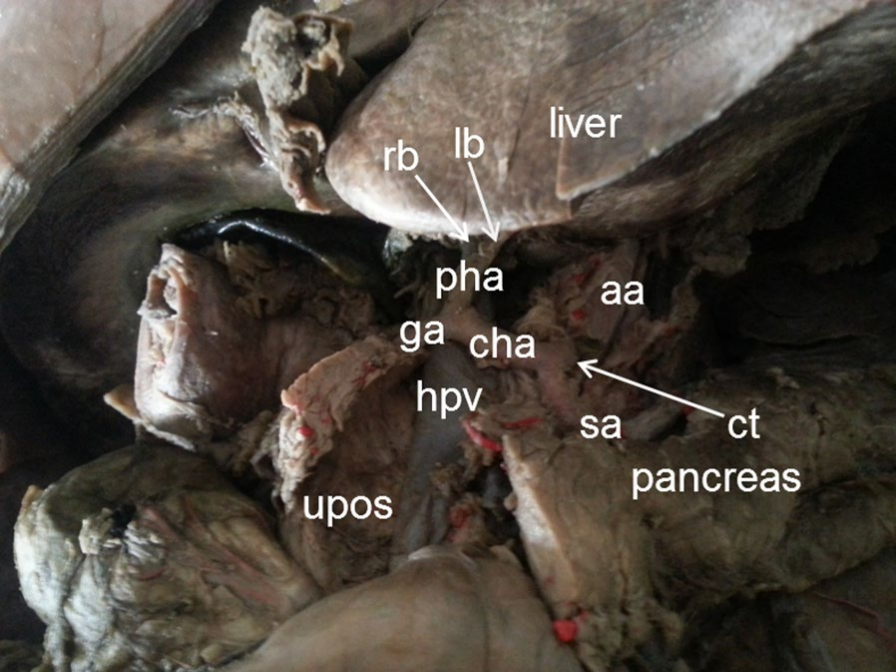
A celiac trunk and its branches. *rb* right branch; *lb* left branch; *pha* proper hepatic artery; *ga* gastroduodenal artery; *cha* common hepatic artery; *aa* abdominal aorta; *hpv* hepatic portal wein; *sa* splenic artery; *ct* celiac trunk; *upos* uncinate process of pancreas

Pre-pancreatic approach: The pancreatic capsule was detached from the inferior margin of the pancreas, with the pancreas as the center, to sweep away the lymph nodes [14]. At the superior border of the pancreatic tail, in the pre-pancreatic space, the isolated gastric omentum and transverse mesocolon were identified emerging from the splenetic artery, and the right gastroepiploic vessels were located inside the omentum posterior to the antrum of the stomach. The stomach body was flipped upward to the right, with the left gastric vessels observable by dissection of the gastropancreatic fold between the pancreatic body and the lesser gastric curvature. At the root of the left gastric artery (i.e., the coronary artery) lay the celiac trunk, with the abdominal aorta and its branches visible at its root (Fig. 2). Along the celiac trunk, the common hepatic artery running to the right could be traced, as well as the common splenetic artery running to the left. The common hepatic artery branched into the proper hepatic artery running toward the first porta hepatis and the gastroduodenal artery heading toward the pancreatic head, the former then bifurcating into the right and left hepatic arteries after giving off the right gastric artery at the hepatoduodenal ligament. Meanwhile, the splenetic artery emanating from the celiac trunk descended to the left and gave off the dorsal pancreatic artery at the pancreatic neck, the right branch of which extended toward the pancreatic head, while the left branch entered the pancreas. The splenetic artery followed a course along the superior margin of the pancreas, giving rise to numerous branches supplying the pancreatic parenchyma, which then sent off the greater pancreatic artery supplying the pancreatic parenchyma, travelling to a point one-third of the distance from the left border to the midline of the pancreas. Subsequently, the splenetic artery gave origin to the left gastroepiploic artery and the short gastric artery above and blew to the pancreatic tail artery, before further bifurcating into superior and inferior splenic lobar arteries about 2.3 cm from the splenic hilum. Each splenic artery then gave off several splenic segmental arteries before entering the spleen. The lymph nodes peripheral to the aforementioned arteries could be dissected through the pre-pancreatic space, as well as the splenic hilar lymph nodes superior to the pancreatic body and tail along the splenic artery (including the skeletonized splenic artery).

**Fig. 3:**
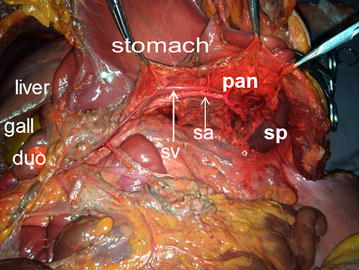
The retro-pancreatic space. *pan* pancreas; *sv* splenic vein; *sa* splenic artery; *sp* spleen; *duo* duodenum

Retro-pancreatic approach: The renal fascia, also known as Gerota’s fascia, encompassed the anterior renal fascia and the posterior renal fascia; together, these fasciae encapsulated the kidney and the suprarenal glands. These two layers of fasciae, which were fused superior to the suprarenal gland, extended to the deepest layers of the retro-pancreatic envelope and connected with the subphrenic fascia. Although there were vessels traveling superior to the mesentery and posterior to the pancreatic neck, no vessels or nerves passed posterior to the pancreatic body and tail or between the two layers of the renal fascia. This verified the safety and convenience of the path that ran posterior to the pancreatic body and the pancreatic tail. The splenocolic ligament was incised along the gastrocolic ligament. The anterior lobe of the mesocolon was dissociated from the inferior margin of the pancreatic body and tail. The splenorenal ligament was also incised, which provided access to the posterior vessel-free area through the inferior margin of the pancreatic body and tail. The pancreatic body and tail were also made accessible, as was the spleen (Fig. 3). The splenic veins were observed as being inferior to the splenic arteries, and their course ran along the dorsal side of the pancreas. The splenic veins received blood from the pancreatic vein branch, the short gastric vein, and the left gastroepiploic vein. The inferior mesenteric vein drained into the splenic vein at about 0.5 cm left of the superior mesenteric vein and posterior to the pancreatic head, and the inferior mesenteric vein joined with the splenic vein at the posterior pancreatic head to form the portal vein. The retro-pancreatic approach was ideal for dissecting the cadavers, as this was the best method for cleaning the lymph nodes peripheral to the splenic veins as well as those located at the splenic hilum inferior to the pancreatic tail. Before the splenic hilum was reached and the lymph nodes inferior to the pancreatic tail were eradicated, the lymph nodes of the pancreatic vein were removed from the center of the tail along the splenic vein, a procedure known as simultaneous skeletonization of the splenic vein.

Consequently, from the perspective of cadaveric anatomy, the lymph nodes peripheral to the pancreatic artery and those located at the splenic hilum superior to the pancreatic tail could be cleaned along the splenic artery in the pre-pancreatic space, while the lymph nodes peripheral to the pancreatic vein and those located at the splenic hilum inferior to the pancreatic tail might be swept away along the splenic vein in the retro-pancreatic space.

### The surgical procedures of live patients

**Fig. 4:**
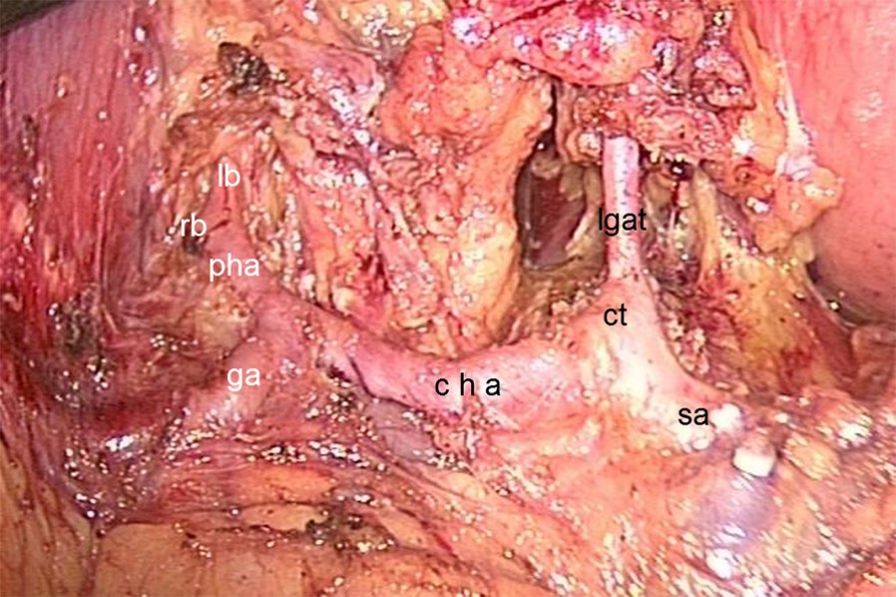
The common hepatic artery and the splenic artery. *lb* left branch; *rb* right branch; *pha* proper hepatic artery; *ga* gastroduodenal artery; *cha* common hepatic artery; *lgat* left gastric artery; *ct* celiac trunk; *sa* splenic artery

With regard to the pre-pancreatic approach, the gastrocolic ligament was first cut off by ultrasound scalpel, and the anterior lobe of the mesocolon was detached up to the inferior margin of the pancreas. The pancreatic capsule was peeled off to the superior margin of the pancreas, adjacent to the short pancreatic vessels. The left gastric-omental vessel was isolated and severed at its root, and thereby eradicating the No. 4sb lymph nodes. Next, the scalpel was moved upward to snip the short gastric vessels and remove the No. 2 and No. 4sa lymph nodes, also incising the left trunk of the vagus nerve beside the esophagus. Then, the gastric omentum and transverse mesocolon were detached at the inferior antrum of the stomach, exposing the gastroduodenal artery and right gastroepiplotic artery, which was then skeletonized and cut off at its end. This allowed the No. 6 and No. 4d lymph nodes to be dissected. Subsequently, the stomach was lifted upward to the right, and the anatomy of the gastropancreatic fold between the pancreatic body and the lesser gastric curvature revealed the left gastric vessel, at the root of which, after isolation, the celiac trunk and its branches, the common hepatic artery, and the splenic artery were identified (Fig. 4). After incision of left gastric vessel, the No. 7, No. 9, No. 8a and No. 8p lymph nodes were swept up. Then, left downward traction of the stomach was exercised and the hepatogastric ligament was incised near the liver. The No. 1 and No. 3 lymph nodes were dissected, the right vagus nerve beside the esophagus being severed at the same time. The dissection was then advanced downward for incision of the superficial layer of the hepatoduodenal ligament and ample dissociation of the hepatic artery, common bile duct, portal vein, and right gastric vessel, allowing removal of the No. 12a, No. 12b and No. 12p lymph nodes. The incision of the right gastric vessel was performed to wipe off the No. 5 lymph nodes. The dissection was continued to cut off the stomachus pyloricus and lower segment of the esophagus by closer linear cutting (Echelon 60 Endopath Stapler; Ethicon Endo-Surgery, Guaynabo, Puerto Rico) and to remove tumor and lymphatic tissues. The pneumoperitoneum was reconstructed and the splenic artery was dissociated at the superior margin of the pancreas. It should be noted that, since the walls of the blood vessels are quite thin, especially the walls of the veins, the nonfunctional side of the ultrasound scalpel should stick close to the blood vessel walls for dissociation of the blood vessels to avoid blood vessel injuries that may precipitate massive hemorrhaging. The branch of the splenic artery supplying the pancreatic parenchyma was prudently detached and severed to the splenic hilum, where the lymphatic tissues superior to the splenic artery were cleared. Thereafter, the surgery turned to the retro-pancreatic approach.

**Fig. 5:**
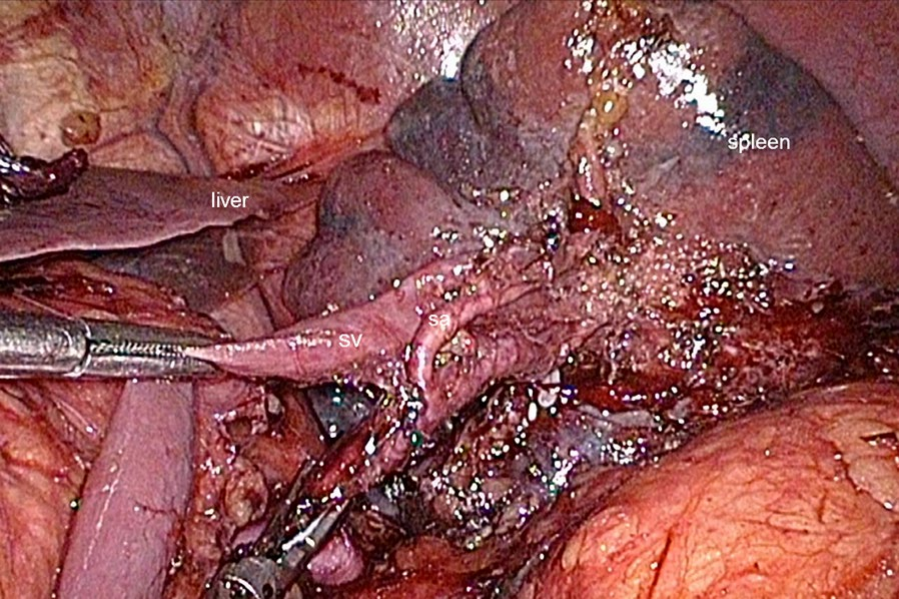
The splenic artery and vein. *sv* splenic vein; *sa* splenic artery

Using the retro-pancreatic approach, the splenocolic ligament and the splenorenal ligament were incised, and the inferior margin of the pancreatic body and tail was isolated anterior to the upper pole of the kidney and the suprarenal fascia. The pancreatic body and tail were gently lifted to the right to expose the splenic vein. The splenic vein was then detached from the center of the pancreatic tail and the pancreatic veins. Fluids were prevented from draining into the splenic vein, with specific effort made to prevent damage to the inferior mesenteric vein. At the splenic hilum, the lymphatic tissues inferior to the pancreatic tail were removed. The splenic artery and vein were completely skeletonized (Fig. 5), thereby completing the dissection of the No. 10, No. 11p, and No. 11d lymph nodes. Finally, the distal esophagus and jejunum were operated on using the Roux-en-y anastomosis technique. The entire procedure was successfully completed.

### Clinicopathological parameters for the patients

Seven males and three females, with an average age of 58.3 ± 17.6 years (39–76 years old), were included in this study. The mean operation time was 243.6 ± 45 min (Table 1). The hemorrhage volume was 232 ± 80 ml (Table 1). The length of hospital stay was approximately 11.5 ± 3 days (Table 1). Generally, the operations proceeded without complications, with only one patient suffering a 1 cm laceration and hemorrhage in the splenic capsule during the dissection of the splenic hilar lymph nodes. This hemorrhage was stopped using electrocautery and gauze. At the time of follow-up (median of 12 months), it was found that none of the patients had died or experienced recurrent or metastatic disease.

## Discussion

Pancreato splenectomy traditionally required the complete removal of LNs in the splenic hilar area to provide a normative D2 LN dissection. However, this method not only failed to improve survival rates among patients [7, 13, 15–17] but also increased mortality rates and postoperative complications. These complications included distinctly higher incidences of pancreatic fistula, acute pancreatitis, abdominal abscess, postoperative diabetes, and postoperative hemorrhages. To prevent some of these complications, surgeons began to adjust dissection procedures and focus on pancreas-preserving dissection of the total stomach and spleen [18]. However, recent reports suggest that spleen dissection may not improve patient survival rates [19, 20]; on the contrary, total spleen dissection may actually cause complications, such as compromising the immune system, spleen heat, blood loss, and portal thrombosis. Therefore, the standardized recommended procedures for open surgery to treat gastric cancer are as follows: subsequent dissection of the gastrocolic ligament, the splenocolic ligament, and the splenorenal ligament and dissociation of the anterior lobe of the mesocolon to the inferior margin of the pancreas. These standardizations also include skeletonization of the splenic artery and vein; adjustment of the pancreatic body, the pancreatic tail, and the spleen through the incision via the retro-pancreatic space; and removal of the splenic hilar lymph nodes using pre-pancreatic and retro-pancreatic approaches that aim to preserve the pancreas and spleen.

Advances in laparoscopic techniques have led to a decrease in the use of open surgery. Laparoscopy initially focused exclusively on abdominal detection, but it was then expanded to include cholecystectomy and colorectal resection, after which it was utilized for conventional resection of the stomach, spleen, and pancreas. Based on these developments, this paper proposes that laparoscopic technology can achieve effects equal to those achieved with the aforementioned open surgery to treat gastric cancer. Because laparoscopic surgeries do not allow for the spleen, pancreatic body, and tail to be removed through the incision, some doubts persist regarding the removal of splenic hilar lymph nodes using pre-pancreatic and retro-pancreatic approaches. Nevertheless, the cadaveric anatomy presented in this paper indicates the existence of a relatively large space anterior to the pancreas, which is ideal for laparoscopic surgery, as well as a similarly convenient normal inter-fascia space free of vessels and nerves. Based on the cadaveric anatomy observed in this study, the researchers who conducted this study successfully conducted ten dissections of splenic hilar lymph nodes using pre-pancreatic and retro-pancreatic approaches, and in doing so, we were able to preserve the pancreases and spleens of all ten patients.

Some surgeons advise *en bloc* resection of gastric tumors, peripheral lymphatic tissues, and splenic hilar lymphatic tissues. However, this technique makes surgery more difficult. When removing the lymph nodes peripheral to the splenic vein by flipping the pancreatic body and tail, improper traction and exposure are likely to cause lacerations of the splenic vein, creating massive hemorrhages. Therefore, this study suggests that tumors and peripheral lymphatic tissues be removed prior to the dissection of splenic hilar lymphatic tissues. This suggested procedural change not only broadens the space for operations and reduces technical difficulties but also facilitates the management of emergencies (such as laceration of the splenic vein).

This procedure is ideal for patients without distal metastasis or obvious fibrosis of enlarged splenic hilar lymph nodes. If the preoperative examination identifies direct extension of the tumor to the spleen and distal pancreas, or if it shows definite LN metastasis at the splenic hilum, then dissection of the entire stomach and spleen is preferable. This is primarily due to the high incidence of postoperative complications and increased mortality rates.

There were some limitations to this study. The sample size was small, no control group was used, and long-term follow-ups were not conducted. However, the preliminary findings from this study demonstrate that for the treatment of proximal gastric cancer, laparoscopic spleen-preserving dissection of the hilar lymph nodes using pre-pancreatic and retro-pancreatic approaches is technically feasible. The study also indicates that this procedure is easy to master and could easily be adopted on a wide scale. However, multicenter, randomized controlled trials that evaluate the long-term outcome of this procedure are necessary to establish the technique’s effectiveness as a cure for proximal gastric cancer.
